# Telomeric replication stress: the beginning and the end for alternative lengthening of telomeres cancers

**DOI:** 10.1098/rsob.220011

**Published:** 2022-03-09

**Authors:** Robert Lu, Hilda A. Pickett

**Affiliations:** Telomere Length Regulation Unit, Children's Medical Research Institute, Faculty of Medicine and Health, University of Sydney, Westmead, NSW 2145, Australia

**Keywords:** telomere maintenance, replication stress, alternative lengthening of telomeres, ALT

## Abstract

Telomeres are nucleoprotein structures that cap the ends of linear chromosomes. Telomeric DNA comprises terminal tracts of G-rich tandem repeats, which are inherently difficult for the replication machinery to navigate. Structural aberrations that promote activation of the alternative lengthening of telomeres (ALT) pathway of telomere maintenance exacerbate replication stress at ALT telomeres, driving fork stalling and fork collapse. This form of telomeric DNA damage perpetuates recombination-mediated repair pathways and break-induced telomere synthesis. The relationship between replication stress and DNA repair is tightly coordinated for the purpose of regulating telomere length in ALT cells, but has been shown to be experimentally manipulatable. This raises the intriguing possibility that induction of replication stress can be used as a means to cause toxic levels of DNA damage at ALT telomeres, thereby selectively disrupting the viability of ALT cancers.

## Graphical abstract

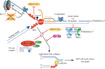

## 1. Introduction

Telomeres are evolutionarily conserved G-rich sequences at the distal ends of linear chromosomes. In humans, telomeres consist of tandem arrays of (TTAGGG)_n_ repeats that are typically 4–8 kb in length and terminate in a 100–300 base pair single-stranded (ss) G-rich 3′ overhang [1–4]. The telomere overhang is able to loop back on itself and strand-invade upstream repeats on the same telomere to form a lariat structure called a telomere-loop (t-loop) [5–7]. Telomeric DNA is bound by the shelterin protein complex, which comprises TRF1, TRF2, RAP1, TIN2, TPP1 and POT1 [8–10], and it is this telomeric DNA and shelterin nucleoprotein structure that protects the ends of the chromosomes from being recognized as sites of DNA damage. Shelterin fulfils a specialized role in the maintenance of telomere integrity and is vital for the fidelity of telomere replication, telomeric transcription and coordinating the deposition of heterochromatic nucleosomes at telomeres [11–17].

Telomere length shortens with cellular division and negatively correlates with chronological age due to limitations in the ability of the replication machinery to fully replicate the linear DNA molecule [18–23]. Telomere shortening eventually results in telomere dysfunction, which is attributed to inadequate shelterin binding and the inability to form a t-loop, and culminates in cellular senescence or apoptosis [6]. These proliferative barriers can be overcome by stabilization of the genome through activation of a telomere maintenance mechanism (TMM). Telomere maintenance is a hallmark of cancer cells and is essential for replicative immortality [24]. Cancer cells typically activate either the ribonucleoprotein enzyme telomerase, or the alternative lengthening of telomeres (ALT) pathway. Importantly, most normal human somatic cells lack a TMM [25].

Telomeres present an exceptional challenge to the replication machinery. This is the cumulative result of the terminal repetitive G-rich sequence content of telomeres, the necessity for constant structural remodelling of telomeric DNA into t-loops, displacement loops (D-loops), DNA/RNA hybrids and G-quadruplex structures during replication and transcription, the constitutive heterochromatic organization of telomeric chromatin, and the engagement of opposing telomere length regulation mechanisms. This is further compounded by a reliance on subtelomeric origins of replication. The replication challenge is heightened at ALT telomeres, with ALT cells displaying exacerbated levels of telomere replication stress that functionally perpetuate telomere extension. In this review, we discuss telomeric replication, the homeostasis between replication stress and repair at ALT telomeres, and how tipping this balance can impact ALT activity and ALT cell viability.

## 2. Telomere replication

DNA replication is a spatially and temporally regulated process that commences in late mitosis and G1 with replication origin licensing, followed by origin activation at the G1–S transition, and origin firing in S-phase [26]. The origin recognition complex (ORC) loads minichromosome maintenance replicative helicase complex (MCM2-7) onto origins [27,28], enabling replication forks to travel in opposite directions from the origin of replication. Some origins remain dormant, with the opportunity to become activated in response to localized fork stalling. Mammalian telomeres, unlike yeast telomeres, are replicated throughout S-phase and lack a consensus replication origin sequence [29]. Telomere replication predominantly originates from subtelomeric regions, with the replisome travelling through the telomeres in a unidirectional manner [13,30]. More recently, it has been revealed that the shelterin component TRF2 can recruit ORC proteins to facilitate replication of telomeres [31,32]. These events are, however, relatively rare and appear to become activated in situations of replication stress as a rescue mechanism to complete telomere replication [13]. The paucity of origins within telomeres means that if a telomeric replication fork encounters a problem, it is unlikely to be rescued by a converging distal fork travelling in the opposite direction. This results in a heightened reliance on fork repair and restart mechanisms at telomeres.

In addition to the lack of replication origins within telomeres, replication of the ends of linear DNA molecules is problematic due to limitations in accessibility by the replication machinery. Specifically, progression of the replication fork requires the coordination of both continuous leading-strand and discontinuous lagging-strand synthesis at the replication fork, the bulk of which is performed by DNA polymerases *ε* and *δ* [33–35]. The ends of the Okazaki fragments produced by lagging-strand synthesis are then processed to remove the RNA primer and ligated together to form a continuous strand. When the replication machinery reaches the end of the chromosome, positioning of the last Okazaki fragment approximately 70 bp upstream on the lagging-strand leads to an inability to completely replicate the lagging-strand template, a limitation known as the end-replication problem [36]. This results in a gradual loss of telomeric sequences with each round of DNA replication and cell division. Additional post-replicative nucleolytic processing of the telomere 3′-overhang by Apollo, ExoI and other factors also contributes to telomere attrition [37–40].

## 3. Telomeres are prone to replication stress

**Figure 1.**
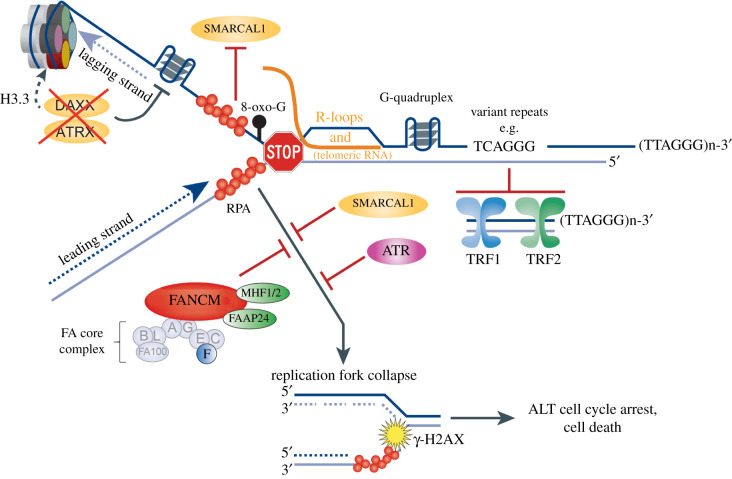
Sources of replication stress at ALT telomeres and key factors that prevent replication fork collapse. Replication stress is exacerbated at ALT telomeres by the loss of ATRX/DAXX function, associated aberrant H3.3 heterochromatin deposition, telomeric R-loops, 8-oxo-G (8-oxo-guanine) lesions and G-quadruplex accumulation, and the interspersion of variant telomere repeats that displace shelterin binding and promote recombination. FANCM and its associated binding partners MHF1/2 and FAAP24, and the FA core complex component FANCF, as well as the SMARCAL1 fork translocase and ATR kinase, protect ALT telomeres from excessive replication stress that can lead to fork collapse. This can then lead to a DNA‐damage response typified by accumulation of replication protein A (RPA) and phosphorylated histone variant H2AX (γ‐H2AX).

Inherent limitations of the replication machinery are compounded by the structural features of telomeres that make them prone to replication stress. Replication stress refers to a slowing or stalling of the replication machinery and occurs when one of the DNA polymerases encounters either a block on the template DNA, or chemical interference such as nucleotide pool depletion or polymerase inhibition [41]. The cellular response to replication stress is orchestrated by the ATR-CHK1 kinase pathway [42]. Telomeres are particularly susceptible to replication stress due to their terminal location, G-rich repetitive sequence content, heterochromatic conformation and the low distribution of telomeric origins [26,43–45]. D-loop and t-loop structures must be unwound to enable replication fork progression. In addition, the G-rich telomeric strand has a high propensity to form four-stranded G-quadruplex structures [46,47]. Removal of G-quadruplexes and unwinding of the t-loop are essential to prevent fork stalling and telomere loss during replication (figure 1). This is performed non-redundantly by several helicases that recognize and unwind G-quadruplexes, including BLM, WRN and RTEL1, FANCJ and ATRX [48,49]. The G-rich strand can also accumulate 8-oxo-G oxidative lesions, which can result in replication defects and telomere loss (figure 1) [50–53].

Transcription–replication conflicts also contribute to replication stress. Telomeric repeat-containing long noncoding RNA, or TERRA, which comprises UUAGGG repeats transcribed terminally from the subtelomere, can form RNA–DNA hybrids and R-loops at telomeres (figure 1) [54]. These hybrid molecules are regulated by RNase-H and several DNA/RNA-helicases, including RTEL1, FANCM, PIF1, as well as the mRNA-transcription export machinery [55–60].

Replication stress is associated with late replication that persists through G2-phase into mitosis, and manifests as mitotic DNA synthesis (MiDAS) [61–63]. Telomeric MiDAS can be induced using a variety of replication blocking agents, including aphidicolin, which inhibits DNA polymerase *α*, and G-quadruplex stabilizing ligands such as PhenDC3, TmPyP4 and Pyridostatin [64–66]. These obstacles highlight the importance of helicases, endonucleases and homology-directed repair factors for telomere replication. Telomeric MiDAS is typically observed on metaphase spreads after nucleoside labelling during mitotic release from G2 arrest [63]. Specifically, it is visualized as telomere synthesis occurring on a single chromatid arm and is indicative of conservative DNA synthesis resulting from a telomere DNA double-strand break (DSB) caused by a collapsed replication fork [67]. Most repair factors required for genomic MiDAS, such as POLD, BLM and RAD52, are also required for telomeric MiDAS, with the exception of MUS81 [61,62,68,69].

## 4. Shelterin protects telomeres from replication stress

Despite these limitations, telomeres are successfully replicated each time a cell divides. This can be predominantly attributed to shelterin, which binds specifically to the telomere sequence and structure, coordinating the recruitment of proteins that maintain replication fidelity. TRF1 and TRF2 display high-affinity binding to double-stranded telomeric DNA through their Myb/SANT domains, while POT1 binds to single-stranded telomeric DNA through its oligonucleotide/oligosaccharide-binding folds [10,70,71]. TRF2 is also capable of binding to branched DNA structures through its N-terminal basic domain [72–74]. In this capacity, shelterin facilitates generation of the 3′ overhang and formation of the t-loop structure, which is stabilized when the 3′ terminus of the telomere is sequestered in a D-loop [6,7,75]. TRF2 also stabilizes and dynamically releases the t-loop structure during replication. This process involves a phospho-switch mechanism that coordinates RTEL1 accessibility during S phase to allow t-loop unwinding and replication, and the release of RTEL1 outside of S phase to enable the t-loop to be restored and maintained [72].

Efficient replication of telomeres is dependent on TRF1, and the absence of TRF1 leads to frequent fork stalling and the appearance of fragile telomeres [76,77]. Fragile telomeres, resulting from replication stress, are often associated with late-replicating intermediates that appear as ultra-fine anaphase bridges. TRF1 also functions to preserve the telomeric chromatin environment by preventing recruitment of homologous recombination factors SMC5/6, BRCA1 and POLD3 that would otherwise promote the engagement of homology-directed repair pathways at the telomeres [78]. Telomere replication navigation further relies on the ability of TRF1 and TRF2 to recruit RTEL1, BLM and WRN helicases to unwind replicative obstacles such as G-quadruplexes [76,79–83], indicative of the prioritization of mechanisms to avoid telomere replication stress.

## 5. Telomere maintenance is a replication stress response

Replication-associated telomere shortening can be counteracted by activation of a TMM, and telomere length maintenance can stabilize the genome. Both telomerase and ALT mechanisms respond, and are recruited, to stalled replication forks in telomeres, indicative of telomere maintenance being a form of DNA repair localized to chromosome ends.

Telomerase is a ribonucleoprotein enzyme comprising the hTERT reverse transcriptase and the hTR RNA component that synthesizes telomeric repeats directly onto the chromosome ends. Telomerase is constitutively bound by four H/ACA ribonucleoprotein-binding factors, dyskerin, NOP10, GAR1 and NHP2, and the Cajal Body (CB) chaperone, TCAB1, which collectively confer telomerase complex stability and facilitate enzyme assembly [84–86]. Telomerase is recruited to telomeres during S to late G2-phase, through a direct interaction between TPP1 and the telomerase essential N-terminal (TEN) domain of hTERT [87–92]. Loss of TRF1 or the presence of stalled forks increases telomerase recruitment to telomeres in an ATR-dependent manner [93]. More recently, it has been shown that telomeric replication stress caused by POT1 dysfunction enables telomeres to travel along nuclear actin filaments to the nuclear periphery, resulting in MiDAS at telomeres in telomerase-positive cells [94,95]. Consistent with replication stress-induced telomere lengthening, POT1 mutations have been identified in a growing number of cancer types, including chronic lymphocytic leukaemia (CLL), melanoma and sarcoma, and are associated with longer telomere lengths [96]. These discoveries demonstrate the capacity for localized replication stress to promote the repositioning of telomeres and the engagement of telomerase.

ALT is defined as telomere maintenance in the absence of telomerase activity [97,98]. While the precise trigger for ALT activation remains unclear, the underlying pathway of ALT-mediated telomere extension has been well characterized and involves DNA repair synthesis mechanisms that are analogous to break-induced replication [99–101]. Specifically, telomere extension events initiate from DSBs that form from exacerbated and unresolved replication stress and collapsed forks that are particularly prevalent in ALT telomeres. Telomere extension can occur in both G2 and prometaphase, is dependent on the BLM-TOP3A-RMI (BTR) complex and the RFC-PCNA-Pol*δ* replisome, and proceeds by both RAD52-dependent and RAD52-independent pathways [63,67,102,103]. Paradoxically, aborted telomere crossover events in the absence of telomere synthesis also contribute to the ALT mechanism, and involve the SLX1-SLX4, MUS81-EME1, XPF-ERCC1 (SMX) endonuclease complex [99]. The balance between telomere crossover and non-crossover extension events dictates overall telomere length and the prevalence of the various ALT phenotypic biomarkers, including extrachromosomal telomeric repeats, ALT-associated promyelocytic leukaemia bodies and telomere sister-chromatid exchange (T-SCE) events.

Telomerase and ALT are predominantly activated in a mutually exclusive manner in cancer cells; however, it is unclear when and why activation of one pathway is favoured over the other [104]. Both telomerase and ALT appear to be regulated by telomeric replication stress, but the specific type of replication stress, the nature and magnitude of the response, and the downstream repair pathways that become activated to confer telomere maintenance, are entirely distinct. The involvement of telomere replication stress suggests that telomere maintenance can be regulated by modifying replication stress within telomeres.

## 6. Cells with alternative lengthening of telomeres display heightened levels of telomeric replication stress

ALT cells display high levels of telomeric DNA damage that arise stochastically as a result of replication stress [15,105]. This is an important and fundamental distinction of ALT telomeres. The underlying reasons for the heightened levels of replication stress at ALT telomeres include interspersed telomeric variant repeats that disrupt the canonical repeat array [106,107], dysregulated telomeric chromatin and an altered nucleoprotein structure resulting from enhanced nuclear receptor binding, reduced shelterin binding and diminished histone levels, all of which exaggerate the inherent problems that normal telomeres present to the replication machinery [108–110].

Telomere variant repeats differ from the canonical TTAGGG sequence at a single nucleotide and include the variants TCAGGG, TGAGGG, TTGGGG and CTAGGG. Variant repeats are enriched in the proximal regions of human telomeres [111–113]. The mismatch repair machinery functions to minimize recombination events between homeologous sequences by promoting heteroduplex rejection. However, in ALT cells, break-induced telomere synthesis appears to occur to some extent within these proximal degenerate regions and can be dramatically enhanced by deletion of the MutS*α* mismatch repair complex [114]. This enables the spread and interspersion of variant repeats throughout the telomeres [106,107] and is presumably an inevitable consequence of the exacerbated DNA damage response at ALT telomeres. Variant repeats have a lower binding affinity for TRF2 and preferentially support binding of NR2C/F orphan nuclear receptor proteins, including TR2, TR4, COUP-TF1 and COUP-TF2 [106,108,113,115,116]. A direct correlation between telomere variant repeat content and NR2C/F orphan nuclear receptor binding is lacking. Nevertheless, nuclear receptors are enriched specifically at ALT telomeres and recruit the NuRD-ZNF827 chromatin remodelling complex, which functions both to preserve the telomeric chromatin and to recruit HR proteins. While the precise ramifications of variant repeat interspersion and nuclear receptor binding are unclear, disruption of shelterin binding at ALT telomeres has the potential to promote telomeric replication stress.

Induction of telomere replication stress in ALT cells has further been attributed to loss of function of the ATRX/DAXX chromatin remodelling complex. Loss of function mutations in ATRX or DAXX are the most common genetic feature of ALT cells [117,118]. ATRX is an ATP-dependent helicase and a member of the sucrose non-fermenting (SNF2) family of chromatin remodellers, while DAXX specifically recognizes and loads H3.3 repressive histone variants at telomeres and pericentric heterochromatin in a replication-independent chromatin assembly pathway [119]. ATRX facilitates telomere replication by maintaining the telomeric heterochromatin, and in its absence, secondary structures such as G-quadruplexes persist, promoting replication fork stalling and collapse, and the engagement of break-induced telomere synthesis (figure 1) [120]. ATRX loss also promotes telomere cohesion and compromises cell cycle regulation of TERRA, causing replication protein A (RPA) to persist at telomeric foci after DNA replication [121,122]. While reintroduction of ATRX into ALT cells causes a reduction in replication fork stalling and an overall repression of ALT, loss of ATRX/DAXX is not sufficient to induce ALT, and it remains unclear what additional activating changes are required for ALT [123].

Depletion of the anti-silencing factor 1 (ASF1) paralogues ASF1a and ASF1b that function as histone chaperones for H3.1/H3.3-H4 dimers during nucleosome assembly simultaneously causes rapid induction of ALT phenotypes and repression of hTERT transcription, indicative of defective histone management resulting in replicative stress and ALT engagement [124]. It has previously been shown that ALT telomeric chromatin is less compacted and heterochromatic when compared to telomerase-positive telomeric chromatin [110]. More recently, it has been demonstrated that ALT activity requires a degree of H3K9 trimethylation, mediated by SETDB1, in order to facilitate the recruitment of recombination factors [125]. The reliance of telomeres on SETDB1 histone methyltransferase activity provides a distinction from pericentric heterochromatin, which uses SUV39H for the deposition of H3K9me3 [125].

ALT cells typically display elevated levels of TERRA and abundant telomeric R-loops. It has been proposed that R-loop formation promotes break-induced telomere synthesis at ALT telomeres by exacerbating telomeric replication stress (figure 1) [126]. Overall, ALT telomeres balance high levels of replication stress with homology-directed repair to maintain a structural and functional equilibrium. This poses the question of what happens when this balance is tipped.

## 7. The cellular implications of replication stress for alternative lengthening of telomeres activity

The implications of replication stress are severe, including DNA damage and genomic instability. The high levels of replication stress at ALT telomeres mean that ALT cells are exquisitely sensitive to further replicative insult or impairment of the replication stress response pathways. This has been comprehensively demonstrated in the context of both FANCM and SMARCAL1 deficiency.

FANCM is an ATPase and DNA translocase that is required for inter-strand cross-link repair, dismantling R-loops and promoting fork restart in response to replication stress [127–129]. FANCM contains a DEAD/DEAH helicase ATPase domain with translocase activity, an expanded C-terminal region that includes a C-terminal nuclease-dead endonuclease ERCC4-like domain and an expanded middle disordered region comprising the MM1 domain that interacts with the Fanconi Anaemia (FA) core complex and the MM2 domain that interacts with the BLM-TOP3A-RMI (BTR) complex [130,131]. FANCM acts as a platform for the assembly of the FA core complex (FANCA, FANCB, FANCC, FANCE, FANCF, FANCG, FANCL, FAAP20 and FAAP100), which monoubiquitinates the FANCI/FANCD2 heterodimer. Monoubiquitinated FANCD2 then localizes to stalled forks and stabilizes FANCI/FANCD2 heterodimers on dsDNA to unhook the stalled fork and promote homologous recombination and lesion bypass [128,132–138].

It was first reported that depletion of FANCM reduces replication efficiency and induces telomeric replication stress in ALT cells [139]. Subsequent studies by three separate laboratories demonstrated that FANCM depletion causes exacerbated levels of telomere replication stress and damage, activation of ATR signalling and a hyper-ALT phenotype [56,59,139,140]. These outcomes caused a G2 cell cycle arrest and were selectively toxic to ALT cells. The ATPase/translocase activity of FANCM and the interaction between FANCM and the BLM-TOP3A-RMI (BTR) complex, which facilitates replication fork remodelling, were both required to attenuate replication stress at ALT telomeres [56,59,140]. This is indicative of persistent R-loops and unresolved replication stress or fork collapse reaching levels that are ultimately catastrophic to ALT cells (figure 1).

SMARCAL1 is an ATP-dependent annealing helicase that interacts with RPA and functions to stabilize replication forks during DNA damage. SMARCAL1, but interestingly not related SNF2 family members HLTF or ZRANB3, plays a key role in mitigating replication stress at ALT telomeres [141]. Specifically, SMARCAL1 depletion causes stalled replication forks to deteriorate to form DSBs, saturating the capabilities of the DNA repair machinery, resulting in chromosomal fusions and thereby driving genomic instability [142,143].

While both FANCM and SMARCAL1 fork translocases exhibit fork remodelling and branch migration activities, the overall magnitude of phenotypic changes observed following FANCM depletion is considerably greater than for SMARCAL1 depletion. This is likely indicative of FANCM being the principal fork translocase required to prevent fork collapse at distally progressing replication forks in ALT telomeres [30,141]. SMARCAL1 is also less capable of remodelling a replication fork when there is RPA accumulation on the lagging strand, which may further explain the more critical role for FANCM at ALT telomeres (figure 1) [144].

## 8. Replication stress modifiers as novel therapeutic targets for alternative lengthening of telomeres cancers

**Figure 2.**
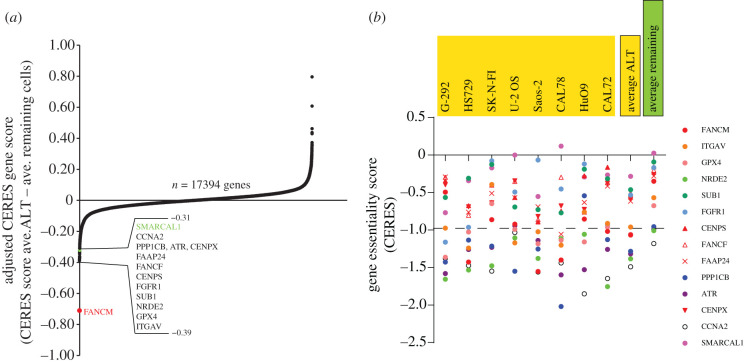
Top 14 gene dependency scores for known ALT cell lines. (*a*) Top 14 ranked adjusted gene essentiality scores from *n* = 17394 genes of eight known ALT cell lines (G-292, HS729, SK-N-FI, U-2 OS, Saos-2, CAL78, HuO9 and CAL72). Adjusted gene scores were calculated from the average gene essentiality score of the eight ALT cell lines minus the average gene essentiality score of all remaining cell lines from the Project Achilles dataset (*n* = 1024, DepMap Public 21Q3). (*b*) Non-adjusted gene essentiality scores (CERES) of each of the eight known ALT cell lines, average CERES scores of all eight ALT cell lines (average ALT) and average CERES scores of all remaining cell lines (average remaining) from the Project Achilles dataset (see https://doi.org/10.6084/m9.figshare.15160110.v2, DepMap 21Q3 Public).

More expansive evidence for the hypersensitivity of ALT cells to replication stress comes from Project Achilles hosted on the Cancer Dependency Map Portal (DepMap Public 21Q3). Project Achilles provides unbiased gene essentiality data derived from genome-wide RNAi and CRISPR/Cas9 loss of function screens performed on over a thousand genomically characterized cancer cell lines. Despite the low prevalence of ALT in this collection of cell lines, and the TMM status of many cell lines remaining unknown, evaluation of ALT-specific gene dependencies demonstrates a clear enrichment of proteins involved in replication fork remodelling and the replication stress response (figure 2*a*). Preferentially essential genes were identified by the average gene essentiality score of eight validated and fully characterized ALT cell lines and were then compared to the average gene essentiality score of all remaining cell lines, the majority of which will be telomerase-positive. FANCM had the top dependency score, being the clear outlier and the major essential gene across all identified ALT cell lines. This demonstrates encouraging convergence between unbiased screening approaches and discovery science in uncovering new and specific cancer dependencies.

FANCM obligate binding partners MHF1/2 (CENPS/CENPX) and FAAP24, which localize FANCM to DNA [59,145], were also enriched in the top 14 preferentially essential genes for ALT (figure 2*b*). Other ALT-dependent genes included FANCF, a key subunit of the FA core complex that interacts directly with FANCM [132], SMARCAL1 and ATR. Overall, this supports the rationale that ALT cells depend on FANCM and, to a lesser degree, the FA pathway, SMARCAL1 and ATR to manage telomeric replication stress, and that deletion or disruption of these proteins is toxic to ALT cells (figures 1 and 2). Several other potential ALT-specific gene dependencies were uncovered, with less explicit roles in replication stress, including ITGAV (Integrin receptor alpha V) and FGFR (fibroblast growth factor receptor 1), which are commonly activated or overexpressed in cancers; GPX4 (glutathione peroxidase 4), which protects cells from membrane lipid peroxidation; NRDE2, which functions in RNA splicing and export; SUB1, a transcriptional co-activator of RNA pol II that has a role in avoiding G4-induced transcriptional damage; and PPP1CB (protein phosphatase 1 catalytic subunit beta) and CCNA2 (Cyclin A2), which are involved in cell cycle progression [146–154]. These data and further analysis and extrapolation of similar resources provide insights into the genes and pathways that are necessary for ALT cancer survival.

The strong biological foundation that supports induction of telomere-specific replication stress as a rapid and potent means to destroy ALT cancer cells implicates proteins such as FANCM and SMARCAL1 as potential targets for the development of ALT therapeutics. Proof of concept for this approach has involved synthetic inhibition of FANCM–BTR complex formation using both a competitive peptide and a small molecule inhibitor [140,145]. Efforts to disrupt the FANCM–BTR interaction as a means to inhibit ALT cancer cell growth are preliminary, but the strategy appears promising. Other functional domains on FANCM may also prove to be viable targets for drug development, for instance the protein–protein interactions between FANCM and FAAP24 and FANCM and MHF1/MHF2, both of which are supported by compelling gene dependency data from Project Achilles, and the ATPase/translocase domain of FANCM [56,59,140,145]. SMARCAL1 may also be a viable therapeutic target, potentially through its HARP or ATPase domains [141]. Other proteins with specific functions in managing telomere replication stress may also emerge as our understanding of the ALT mechanism matures.

## 9. Conclusion

Cancers that rely on the ALT pathway of telomere maintenance constitute approximately 10–15% of all cancers, with this proportion rising substantially in tumours of mesenchymal and neuroepithelial origin [155]. Although ALT cancers are typically aggressive with poor prognosis, ALT status is not yet considered in cancer diagnosis, with no specific treatments currently available for ALT cancers. Cancers that use the ALT pathway have an intrinsic reliance on replication stress to direct DNA repair pathways to the telomeres to achieve homology-directed telomere extension. This reliance confers a heightened sensitivity to the disruption of factors that regulate telomere replication stress in ALT cells. Parallel hypothesis-driven molecular studies and unbiased knock-out screens have identified FANCM and SMARCAL1 as key proteins required for ALT cell viability, with specific roles in managing replication stress at ALT telomeres. Furthermore, the disruption of FANCM function using a small molecule inhibitor has been shown to be selectively detrimental to ALT cancer cells. Despite the strong molecular foundation for the manipulation of replication stress at ALT telomeres having therapeutic potential, substantial effort is required to further develop these discoveries.

Adopting a strategy of catastrophic telomere replication stress induction as a means to destroy ALT cancer cells has obvious pitfalls. As break-induced telomere synthesis events in ALT cells emanate from the deterioration of stalled replication forks, there is a clear risk that exacerbated telomere replication stress will fail to hit toxic levels and will instead promote ALT activity, essentially stoking the fire in an already aggressive tumour environment. Encouragingly, this does not appear to be the case for FANCM inhibition, with cell toxicity occurring rapidly and broadly. Nevertheless, this is an important consideration for future ALT therapeutics. As the potential for ALT targeted cancer therapeutics develops, understanding the origin and type of replication stress that underlies ALT activity, the host of proteins that manage this replication stress, and the toxic outcomes of fork stalling and collapse all require further attention.

## Data Availability

The gene dependency score dataset used in this publication is available at https://depmap.org/portal/download/all/. https://doi.org/10.6084/m9.figshare.15160110.v2. The DepMap Public 21 dataset used for figure 2 is accessible at https://depmap.org/portal/download/.
